# Case Report: Sustained mitochondrial damage in cardiomyocytes in patients with severe propofol infusion syndrome

**DOI:** 10.12688/f1000research.24567.1

**Published:** 2020-07-16

**Authors:** Satoshi Karasawa, Taka-aki Nakada, Naoto Mori, Michiko Daimon, Hideyuki Miyauchi, Tetsuya Kanai, Hiroyuki Takano, Yoshio Kobayashi, Shigeto Oda

**Affiliations:** 1 Department of Emergency and Critical Care Medicine,, Graduate School of Medicine,Chiba University, 1-8-1 Inohana, Chuo, Chiba, 260-8677, Japan; 2 Department of Cardiovascular Medicine, Graduate School of Medicine, Chiba University, 1-8-1 Inohana, Chuo, Chiba, 260-8677, Japan; 3 Department of Neurology, Graduate School of Medicine, Chiba University, 1-8-1 Inohana, Chuo, Chiba, 260-8677, Japan; 4 Department of Molecular Cardiovascular Pharmacology, Graduate School of Pharmaceutical Sciences, Chiba University, 1-8-1 Inohana, Chuo, Chiba, 260-8677, Japan

**Keywords:** mitochondria, arrhythmia, cardiac failure, Propofol

## Abstract

**Introduction:** Propofol infusion syndrome (PRIS) is rare but a potentially lethal adverse event. The pathophysiologic mechanism is still unknown.

**Patient concerns: **A 22-year-old man was admitted for the treatment of Guillain-Barré syndrome. On day six, he required mechanical ventilation due to progressive muscle weakness; propofol (3.5 mg/kg/hour) was administered for five days for sedation. On day 13, he had hypotension with abnormal electrocardiogram findings, acute kidney injury, hyperkalemia and severe rhabdomyolysis.

**Diagnosis and interventions: **The patient was transferred to our intensive care unit (ICU) on suspicion of PRIS. Administration of noradrenaline and renal replacement therapy and fasciotomy for compartment syndrome of lower legs due to PRIS-rhabdomyolysis were performed.

**Outcomes: **The patient gradually recovered and was discharged from the ICU on day 30. On day 37, he had repeated sinus bradycardia with pericardial effusion in echocardiography. Cardiac ^18^F-FDG PET on day 67 demonstrated heterogeneous ^18^F-FDG uptake in the left ventricle. Electron microscopic investigation of endomyocardial biopsy on day 75 revealed mitochondrial myelinization of the cristae, which indicated mitochondrial damage of cardiomyocytes. He was discharged without cardiac abnormality on day 192.

**Conclusions: **Mitochondrial damage in both morphological and functional aspects was observed in the present case. Sustained mitochondrial damage may be a therapeutic target beyond the initial therapy of discontinuing propofol administration.

## Abbreviations

PRIS, propofol infusion syndrome; ^18^F-FDG PET, ^18^F-fluorodeoxyglucose positron emission tomography.

## Introduction

Propofol is extensively used in the intensive care units (ICU) for sedation ^1^. Propofol infusion syndrome (PRIS) is widely recognized as an adverse event of this commonly used drug, but is rare and potentially lethal ^2^. The pathophysiologic mechanism is still unknown. However, mitochondrial damage is suggested to be a potential pathogenesis mechanism. Here we report a severe case of PRIS with evidence of mitochondrial damage in both morphological and functional aspects.

## Case presentation

**Figure 1.**
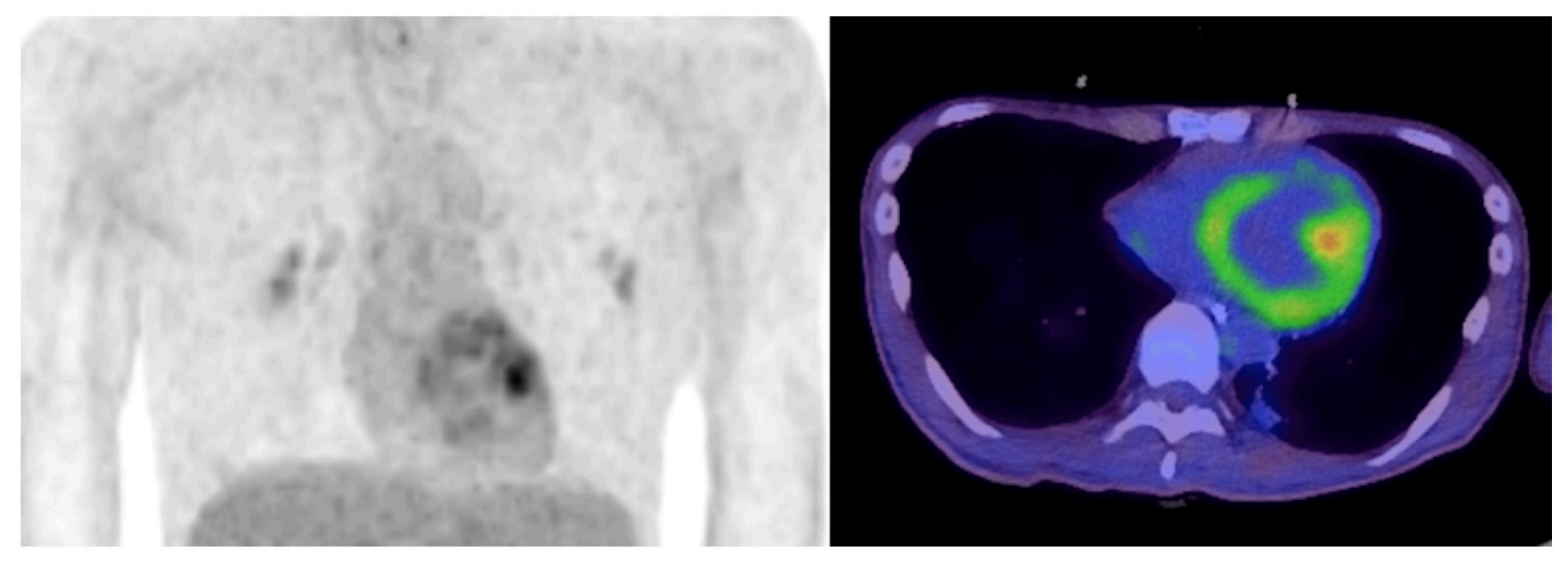
^18^F-fluorodeoxyglucose positron emission tomography. ^18^F-fluorodeoxyglucose positron emission tomography showed heterogeneous ^18^F-FDG uptake in left ventricle.

**Figure 2.**
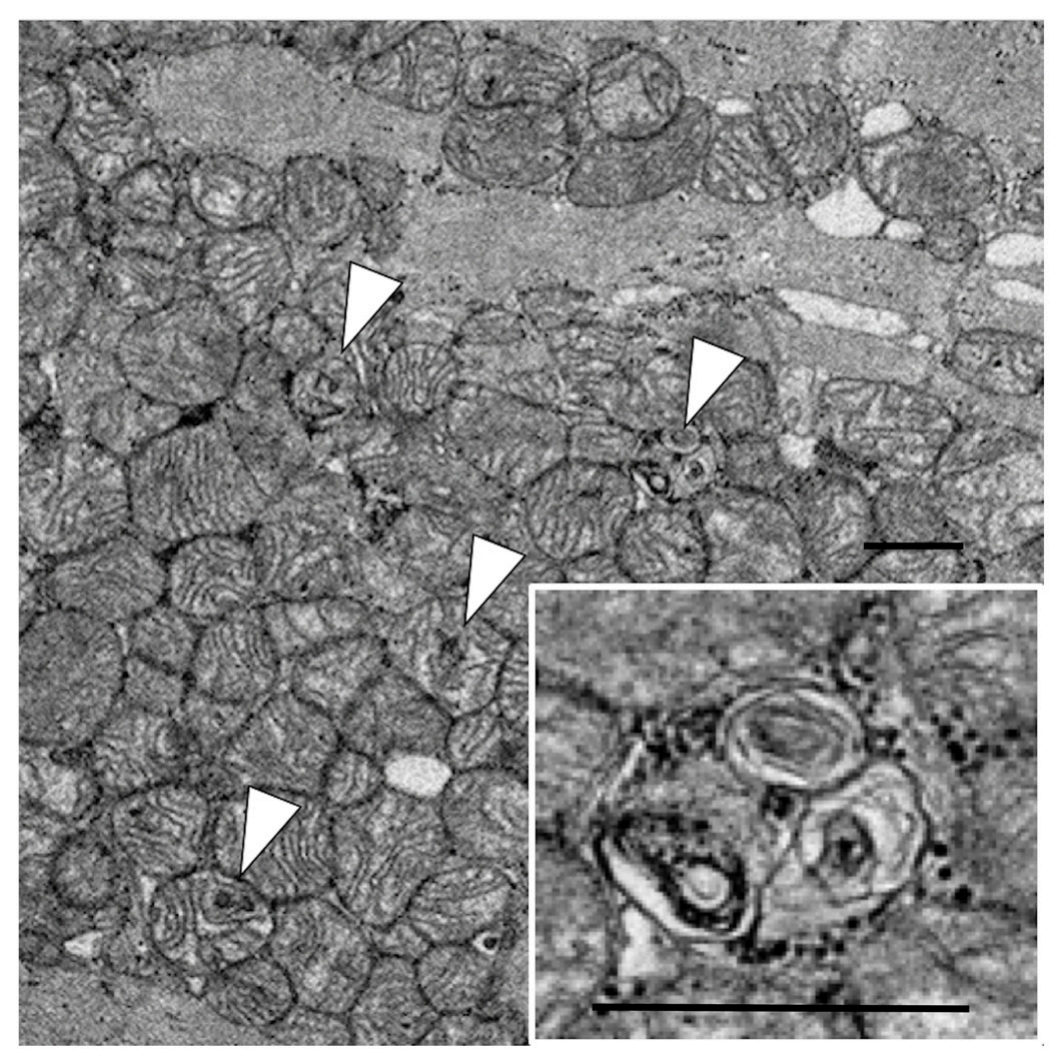
Electron microscopic investigation of endomyocardial biopsy. Electron microscopy revealed mitochondrial myelinizations of the cristae in cardiomyocyte. Arrows indicate cardiomyocytes with the mitochondrial injury. Scale bar: 1 µm.

A 22-year-old man, who was a healthy university student with Japanese ancestry without preexisting medical and family history, experienced muscle weakness and was admitted for the treatment of Guillain-Barré syndrome. On day six, he required mechanical ventilation due to progressive muscle weakness; propofol (3.5 mg/kg/hour) was administered via a peripheral venous catheter for five days for sedation. On day 13, he had hypotension with abnormal electrocardiogram findings (ST elevation in II, III, and aV _F_). Blood test revealed acute kidney injury, hyperkalemia and severe rhabdomyolysis (serum creatinine phosphokinase 271,700 IU/L, normal range 68-287 IU/L). He was transferred to our ICU on suspicion of PRIS by excluding other diagnoses. Administration of noradrenaline via a central venous catheter (0.3 µg/kg/min) and hemodialysis were initiated, and fasciotomy by orthopedic surgeons under general anesthesia without propofol was required for compartment syndrome of lower legs due to PRIS-rhabdomyolysis. Noradrenaline was gradually reduced and terminated on day 15. He gradually recovered from cardiac and renal dysfunction according to echocardiography and blood tests and was discharged from the ICU on day 30. On day 37, he repeatedly presented sinus bradycardia and right bundle branch block in continuous electrocardiogram monitoring, eventually requiring temporary pacing via the intracardiac placement of a pacing wire, with a finding of pericardial effusion on echocardiography. Detailed examination including cardiac ^18^F-fluorodeoxyglucose positron emission tomography ( ^18^F-FDG PET) was conducted to evaluate whether these late-phase cardiac events were related to PRIS. Cardiac ^18^F-FDG PET on day 67 demonstrated heterogeneous ^18^F-FDG uptake in the left ventricle ( Figure 1). Electron microscopic investigation of the endomyocardial biopsy, which was taken on day 75 to examine the cause of cardiac dysfunction, revealed abnormal findings in the mitochondria of the cardiomyocytes, including myelinization of the cristae ( Figure 2). Since weakness of respiratory muscles and extremities muscles needed mechanical ventilation and rehabilitation, he was treated in the hospital for another 3 months．He was taken off the ventilator and transferred to another hospital on day 192 due to persisting muscle weakness, but with normal cardiac function without arrhythmia. Three-year follow-up revealed that he had normal cardiac function with normal activities of daily living.

## Discussion and conclusions

 Mitochondrial damage is suggested as a potential pathogenesis of PRIS ^2– 4^. Mitochondrial damage was observed as a morphological finding in an electron microscopic evaluation of the heart in an autopsy case of PRIS ^5^. Similarly, mitochondrial damage was observed in the endomyocardial biopsy two months after the onset in the present case. Mitochondrial damage can also be detected as a functional impairment of fatty acid utilization with alternatively increased glucose utilization ^6^. The uptake of a glucose analog ( ^18^F-FDG) in left ventricle on day 67 ( Figure 1) in the present case implies a shift in the energy substrate of cardiomyocytes from fatty acid to glucose, suggesting mitochondrial damage. To the best of our knowledge, this is the first to report a case of PRIS with evidence of mitochondrial damage in both morphological and functional aspects, which is the strength of this case report. The evidence of mitochondrial damage by ^18^F-FDG PET and electron microscopic investigation was not repeatedly evaluated during the time-course but a single time-point ( ^18^F-FDG PET on day 67 and endomyocardial biopsy 75), which is a potential limitation. Since the mitochondrial damage was detected 2 month later after PRIS onset, sustained mitochondrial damage may be a therapeutic target beyond the initial therapy of discontinuing propofol administration.

## Data availability

All data underlying the results are available as part of the article and no additional source data are required.

## Consent

Written informed consent for publication of their clinical details and clinical images was obtained from the patient.
