# Supplementary material for: Case Report: Sustained mitochondrial damage in cardiomyocytes in patients with severe propofol infusion syndrome
Source: F1000Res. 2022 Jan 20;9:712. Originally published 2020 Jul 16. [Version 2] doi: 10.12688/f1000research.24567.2 (PMC8905003; doi:10.12688/f1000research.24567.2)
Supplement: Peer review history

# Peer review: Reviewer response for version 2

**DOI:** 10.5256/f1000research.112729.r120539

**Published:** 2022-03-08

**Authors:** Anja Karlstaedt

**Affiliations:** 1 Cedars Sinai Medical Center , Los Angeles, CA, USA

The authors have addressed all my concerns and appropriately revised the manuscript. I can confirm that the submission is at an acceptable scientific standard.

Are enough details provided of any physical examination and diagnostic tests, treatment given and outcomes?

Yes

Is the case presented with sufficient detail to be useful for other practitioners?

Partly

Is sufficient discussion included of the importance of the findings and their relevance to future understanding of disease processes, diagnosis or treatment?

Partly

Is the background of the case’s history and progression described in sufficient detail?

Partly

Reviewer Expertise:

Cardiology, Metabolism, Systems Biology, Computational modeling.

I confirm that I have read this submission and believe that I have an appropriate level of expertise to confirm that it is of an acceptable scientific standard.

# Peer review: Reviewer response for version 2

**DOI:** 10.5256/f1000research.112729.r120540

**Published:** 2022-01-27

**Authors:** Petra Fallier-Becker, Susanne Haen

**Affiliations:** 1 Institute of Pathology and Neuropathology, University Hospital of Tuebingen, Tuebingen, Germany; 2 Institute of pathology and neuropathology, University of Tuebingen, Tuebingen, Germany

The revisions are appropriate.

Are enough details provided of any physical examination and diagnostic tests, treatment given and outcomes?

Partly

Is the case presented with sufficient detail to be useful for other practitioners?

No

Is sufficient discussion included of the importance of the findings and their relevance to future understanding of disease processes, diagnosis or treatment?

No

Is the background of the case’s history and progression described in sufficient detail?

Yes

Reviewer Expertise:

Pathology, Neuroscience, Electron Microscopy

We confirm that we have read this submission and believe that we have an appropriate level of expertise to confirm that it is of an acceptable scientific standard.

# Peer review: Reviewer response for version 1

**DOI:** 10.5256/f1000research.27101.r74771

**Published:** 2020-11-27

**Authors:** Anja Karlstaedt

**Affiliations:** 1 Cedars Sinai Medical Center , Los Angeles, CA, USA

Karasawa *et al*. present a case report titled "Sustained mitochondrial damage in cardiomyocytes in patients with severe propofol infusion syndrome." The authors describe the case of a 22-year-old man of Japanese descent who developed a propofol infusion syndrome (PRIS) as a complication of his treatment of Guillain-Barre syndrome. PRIS is a rare syndrome with an unknown pathophysiologic mechanism, lack of specific signs and symptoms, and high mortality. Together these factors pose a challenge for clinicians to identify patients with potential PRIS and to provide treatment. The present case report may help other clinicians manage a patient with possible PRIS and allow future studies to elucidate the underlying molecular mechanisms causing the syndrome. 

 The authors present their case clearly and concisely. However, the report has several shortcomings, which should be addressed in a revision.

 Major concerns

1. The authors need to provide quantifications for the ^18^F-FDG PET imaging, including how they normalized their counts (e.g., brain, blood glucose level before and after the imaging). The PET image lacks a scale.
2. Additional information regarding the endomyocardial biopsy is required. The authors should comment if they observed apoptotic myocytes, which stages of morphological stages of mitochondrial degeneration they found (e.g., A to D), and potentially provide quantification. 
3. The authors indicate in their introduction that "mitochondrial damage is suggested to be a potential pathogenesis mechanisms." Further, in the discussion and conclusions, the authors suggest that "Mitochondrial damage can also be detected as a functional impairment of fatty acid utilization with alternatively increased glucose utilization [Ref 6]." Readers who are less familiar with PRIS or cardiac metabolism would benefit from a brief description of how propofol impairs carnitine palmitoyl transport activities and cardiac calcium dynamics, potentially affecting the oxidation of fatty acids. The shift in energy-providing substrates is an essential pathophysiological aspect of the case report and, in general, cardiac stress response. Additional descriptions and references will help readers to follow the author's rationale.
4. The authors need to differentiate their case presentation between mitochondrial damage at the structural level as supported by the histology and functional changes due to metabolic remodeling. Metabolic remodeling in the heart can precede structural changes (for reference see: References ^1^^-^^5^). ^18^F-FDG PET imaging shows increased glucose utilization in the heart on day 67, and the authors provide evidence for mitochondrial structural damage at day 75. The authors correctly conclude that these results imply a shift from predominant fatty acid oxidation to glucose. However, the data are insufficient to conclude that these flux changes occur due to mitochondrial damage or pharmacologic modulation of key regulatory enzymes and transporters, without additional time points.
5. The authors state: "The evidence of mitochondrial damage by ^18^F-FDG PET […]." ^18^F-FDG-PET imaging does not determine mitochondrial damage but measures glucose uptake. Please revise (for example: "The evidence of increased glucose uptake and mitochondrial damage by […].")

Minor remarks and questions:

1. Did the authors observe any changes in the blood glucose level or glucose tolerance of the patient?
2. The authors may consider further quantification of mitochondrial degeneration in their tissue sample using markers for autophagy and apoptosis, e.g., BNIP3, FOXO3a, BCL-2, or OPA1.

Are enough details provided of any physical examination and diagnostic tests, treatment given and outcomes?

Yes

Is the case presented with sufficient detail to be useful for other practitioners?

Partly

Is sufficient discussion included of the importance of the findings and their relevance to future understanding of disease processes, diagnosis or treatment?

Partly

Is the background of the case’s history and progression described in sufficient detail?

Partly

Reviewer Expertise:

Cardiology, Metabolism, Systems Biology, Computational modeling.

I confirm that I have read this submission and believe that I have an appropriate level of expertise to confirm that it is of an acceptable scientific standard, however I have significant reservations, as outlined above.

## References

[1] : Oncometabolite d-2-hydroxyglutarate impairs α-ketoglutarate dehydrogenase and contractile function in rodent heart. *Proceedings of the National Academy of Sciences* .2016;113(37) : 10.1073/pnas.1601650113 10436-10441 10.1073/pnas.1601650113 PMC502742227582470

[2] : Glucose 6-Phosphate Accumulates via Phosphoglucose Isomerase Inhibition in Heart Muscle. *Circulation Research* .2020;126(1) : 10.1161/CIRCRESAHA.119.315180 60-74 10.1161/CIRCRESAHA.119.315180 PMC704800431698999

[3] : Metabolic Remodeling Promotes Cardiac Hypertrophy by Directing Glucose to Aspartate Biosynthesis. *Circulation Research* .2020;126(2) : 10.1161/CIRCRESAHA.119.315483 182-196 10.1161/CIRCRESAHA.119.315483 PMC844812931709908

[4] : Glucose promotes cell growth by suppressing branched-chain amino acid degradation. *Nature Communications* .2018;9(1) : 10.1038/s41467-018-05362-7 10.1038/s41467-018-05362-7 PMC606255530050148

[5] : Mammalian target of rapamycin (mTOR) inhibition with rapamycin improves cardiac function in type 2 diabetic mice: potential role of attenuated oxidative stress and altered contractile protein expression. *J Biol Chem* .2014;289(7) : 10.1074/jbc.M113.521062 4145-60 10.1074/jbc.M113.521062 24371138PMC3924280

# Response

**Published:** 2021-12-16

**Authors:** Satoshi Karasawa

**Affiliations:** Chiba University, Japan

Thank you for taking your time to review and comment on our manuscript. Sorry for the delay in response due to COVID-19 pandemic.

1. The authors need to provide quantifications for the 18F-FDG PET imaging, including how they normalized their counts (e.g., brain, blood glucose level before and after the imaging). The PET image lacks a scale.l.

**Response:**

 Thank you for the comment. According to the comment, we revised the sentence and scale.

**Added** **(** **Case presentation** **):**

 Detailed examination including cardiac 18F-fluorodeoxyglucose positron emission tomography (18F-FDG PET) was conducted to evaluate whether these late-phase cardiac events were related to PRIS. Cardiac 18F-FDG PET on day 67 demonstrated heterogeneous 18F-FDG uptake in the left ventricle with the maximum standardized uptake value (SUV) of 3.97. Blood glucose level before and after imaging was 90 mg/dL.

**Added** **(Figure and** **Figure legends):**

 I added scale bar to the Figure1.

 Figure1. 18F-fluorodeoxyglucose positron emission tomography.

 18F-fluorodeoxyglucose positron emission tomography showed heterogeneous 18F-FDG uptake in left ventricle. Scale bar: 5cm

2. Additional information regarding the endomyocardial biopsy is required. The authors should comment if they observed apoptotic myocytes, which stages of morphological stages of mitochondrial degeneration they found (e.g., A to D), and potentially provide quantification.

**Response:**

 Thank you for the comment. According to the comment, we added a sentence in the Case presentation section. Since the sample preparation for electron microscope images could affect the morphological stages, we assume that we could not accurately estimate the morphological stage of mitochondrial degeneration.

**Added** **(** **Case presentation** **):**

 Electron microscopic investigation of the endomyocardial biopsy, which was taken on day 75 to examine the cause of cardiac dysfunction, revealed abnormal findings in the mitochondria of the cardiomyocytes, including myelinization of the cristae ( Figure 2), which was interpreted as mitochondrial damage. In addition, apoptotic myocytes were not observed.

3. The authors indicate in their introduction that "mitochondrial damage is suggested to be a potential pathogenesis mechanisms." Further, in the discussion and conclusions, the authors suggest that "Mitochondrial damage can also be detected as a functional impairment of fatty acid utilization with alternatively increased glucose utilization [Ref 6]." Readers who are less familiar with PRIS or cardiac metabolism would benefit from a brief description of how propofol impairs carnitine palmitoyl transport activities and cardiac calcium dynamics, potentially affecting the oxidation of fatty acids. The shift in energy-providing substrates is an essential pathophysiological aspect of the case report and, in general, cardiac stress response. Additional descriptions and references will help readers to follow the author's rationale

**Response:**

 Thank you for the comment. According to the comment, we added a reference and sentences in the Discussion and Conclusions.

**Added** **(** **Discussion and Conclusions** **): :**

 Propofol is known to inhibit the effects of carnitine palmityl transferase 1 (CPT 1), which transports long-chain fatty acids into the mitochondria ^3^. Thus, propofol potentially impairs carnitine palmitoyl transport activities and cardiac calcium dynamics, affecting the oxidation of fatty acids.

4. The authors need to differentiate their case presentation between mitochondrial damage at the structural level as supported by the histology and functional changes due to metabolic remodeling. Metabolic remodeling in the heart can precede structural changes (for reference see: References 1-5). 18F-FDG PET imaging shows increased glucose utilization in the heart on day 67, and the authors provide evidence for mitochondrial structural damage at day 75. The authors correctly conclude that these results imply a shift from predominant fatty acid oxidation to glucose. However, the data are insufficient to conclude that these flux changes occur due to mitochondrial damage or pharmacologic modulation of key regulatory enzymes and transporters, without additional time points.

**Response:**

 Thank you for the comment. According to the comment, we added sentences in the Discussion and Conclusions.

**Added** **(** **Discussion and Conclusions** **):**

 The evidence of mitochondrial damage by 18F-FDG PET and electron microscopic investigation was not repeatedly evaluated during the time-course but a single time-point (18F-FDG PET on day 67 and endomyocardial biopsy 75), which is a potential limitation. Additional timepoints data in future studies could reveal that shifts from predominant fatty acid oxidation to glucose occur due to mitochondrial damage or pharmacologic modulation of key regulatory enzymes and transporters.

5. The authors state: "The evidence of mitochondrial damage by 18F-FDG PET […]." 18F-FDG-PET imaging does not determine mitochondrial damage but measures glucose uptake. Please revise (for example: "The evidence of increased glucose uptake and mitochondrial damage by […].")

**Response:**

 According to the comment, we revised the sentence.

**Previous** **(** **Discussion and Conclusions** **):**

 The evidence of mitochondrial damage by 18F-FDG PET and electron microscopic investigation was not repeatedly evaluated during the time-course but a single time-point ( 18F-FDG PET on day 67 and endomyocardial biopsy 75), which is a potential limitation. Since the mitochondrial damage was detected 2 month later after PRIS onset, sustained mitochondrial damage may be a therapeutic target beyond the initial therapy of discontinuing propofol administration.

**Revised** **(** **Discussion and Conclusions** **)** **:**

 The evidence of increased glucose uptake by 18F-FDG PET and mitochondrial damage by electron microscopic investigation was not repeatedly evaluated during the time-course but a single time-point ( 18F-FDG PET on day 67 and endomyocardial biopsy 75), which is a potential limitation. Since the mitochondrial damage was detected 2 month later after PRIS onset, sustained mitochondrial damage may be a therapeutic target beyond the initial therapy of discontinuing propofol administration.

Minor concerns

1. Did the authors observe any changes in the blood glucose level or glucose tolerance of the patient?

**Response:**

 According to the comment, we added the sentences.

**Added** **(** **Case presentation** **):**

 Detailed examination including cardiac 18F-fluorodeoxyglucose positron emission tomography (18F-FDG PET) was conducted to evaluate whether these late-phase cardiac events were related to PRIS. Cardiac 18F-FDG PET on day 67 demonstrated heterogeneous 18F-FDG uptake in the left ventricle with the maximum standardized uptake value (SUV) of 3.97. Blood glucose level before and after imaging was 90 mg/dL.

**Added:**

 We observed no significant changes in the blood glucose and glucose tolerance of the patient during his hospital stay.

2. The authors may consider further quantification of mitochondrial degeneration in their tissue sample using markers for autophagy and apoptosis, e.g., BNIP3, FOXO3a, BCL-2, or OPA1.

**Response:**

 Thank you for the comment. Unfortunately, we have no tissue sample available for further measurements.

# Peer review: Reviewer response for version 1

**DOI:** 10.5256/f1000research.27101.r74264

**Published:** 2020-11-12

**Authors:** Petra Fallier-Becker, Susanne Haen

**Affiliations:** 1 Institute of pathology and neuropathology, University of Tuebingen, Tuebingen, Germany; 2 Institute of Pathology and Neuropathology, University Hospital of Tuebingen, Tuebingen, Germany

The concentration of several short-chain acylcarnitines in the patient's blood is missing.

 Furthermore,the authors showed an electron microscopical image of mitochondria in the heart muscle with "myelinization of the cristae" but they don't write anything about the pathological backround, the interpretation is missing. In our article (Vollmer *et al.*) the amorphous figures associated with mitochondria in the heart muscle could be interpreted as free fatty acids but they look completely different than the myelinized structures in the mitochondria in the article of Karasawa *et al.*

Are enough details provided of any physical examination and diagnostic tests, treatment given and outcomes?

Partly

Is the case presented with sufficient detail to be useful for other practitioners?

No

Is sufficient discussion included of the importance of the findings and their relevance to future understanding of disease processes, diagnosis or treatment?

No

Is the background of the case’s history and progression described in sufficient detail?

Yes

Reviewer Expertise:

Pathology, Neuroscience, Electron Microscopy

We confirm that we have read this submission and believe that we have an appropriate level of expertise to confirm that it is of an acceptable scientific standard, however we have significant reservations, as outlined above.

# Response

**Published:** 2021-12-16

**Authors:** Satoshi Karasawa

**Affiliations:** Chiba University, Japan

Thank you for taking your time to review and comment on our manuscript. Sorry for the delay in response due to COVID-19 pandemic. Unfortunately, we have no samples available to measure short-chain acylcarnitines. According to the comments, we added the sentences to improve the manuscript.

**Added (Case presentation):**

 Electron microscopic investigation of the endomyocardial biopsy, which was taken on day 75 to examine the cause of cardiac dysfunction, revealed abnormal findings in the mitochondria of the cardiomyocytes, including myelinization of the cristae (Figure 2), which was interpreted as mitochondrial damage.

**Previous** **(** **Discussion and Conclusions** **):** **:**

 Mitochondrial damage was observed as a morphological finding in an electron microscopic evaluation of the heart in an autopsy case of PRIS ^5 ^. Similarly, mitochondrial damage was observed in the endomyocardial biopsy two months after the onset in the present case.

**Revised** **(** **Discussion and Conclusions** **):**

 Mitochondrial damage was observed as a morphological finding in an electron microscopic evaluation of the heart in an autopsy case of PRIS ^5 ^. We found myelinization of the cristae in cardiomyocyte on day 75; however, similar findings were not observed in postmortem electron microscopical image of mitochondria of PRIS in the previous report ^5^. Different clinical course and timepoints may alter mitochondrial conditions. The autopsy study measured blood levels of short-chain acylcarnitines, while we have no blood sample available for the measurement. Further studies measuring blood levels of short-chain acylcarnitines would strengthen the case results.
